# Relationship between intraoperative blood pressure variability and postoperative acute kidney injury in pediatric cardiac surgery

**DOI:** 10.1007/s00467-025-06659-8

**Published:** 2025-01-27

**Authors:** Rong Xiao, Ming Luo, Hong Yu, Yan Zhang, Feng Long, Weina Li, Ronghua Zhou

**Affiliations:** 1 Department of Anesthesiology, West China Hospital, Sichuan University, No. 37 Guoxue Lane, Wuhou District, Chengdu, 610000 Sichuan China; 2 Department of Anesthesiology, Xichang People’s Hospital, Xichang, 615000 Sichuan China

**Keywords:** Pediatric, Cardiac surgery, Acute kidney injury, Blood pressure variability

## Abstract

**Background:**

Cardiac surgery-associated acute kidney injury (CSA-AKI) is a notably common complication in pediatrics, with an incidence rate ranging from 15 to 64%. This rate is significantly higher than that observed in adults. Currently, there is a lack of substantial evidence regarding the association between intraoperative blood pressure variability (BPV) during cardiac surgery with cardiopulmonary bypass (CPB) and the development of AKI in pediatric patients.

**Methods:**

This retrospective observational study encompassed children aged 0–7 years undergoing cardiac surgery with CPB. Intraoperative BPV was calculated using coefficients of variation (CVs) and the area under the curve (AUC). Univariate and multivariate analyses were employed to identify risk factors associated with CSA-AKI.

**Results:**

Among 570 patients (median age 1 year) reviewed, 36.1% developed CSA-AKI (68.9% risk stage, 22.8% injury stage, and 8.3% failure stage). After adjusting for other variables, male gender (*OR* = 2.044, 95% CI 1.297–3.222, *P* = 0.002), congenital heart surgery risk assessment grade (RACHS-1) classification ≥ 3 (*OR* = 0.510, 95% *CI* 0.307–0.846, *P* = 0.009), longer CPB time (*OR* = 1.022, 95% *CI* 1.007–1.037, *P* = 0.004) and higher peak value of intraoperative vasoactive inotropic score (VIS) (*OR* = 1.072, 95% *CI* 1.026–1.119, *P* = 0.002) were identified as independent risk factors for CSA-AKI. ± 30% AUCm was different in univariate analysis (*P* = 0.014), however, not statistically different in multifactor analysis (*P* = 0.610).

**Conclusion:**

Greater BPV, specifically MAP variations exceeding 30% AUC during CPB, may be a potential risk factor for CSA-AKI in pediatric patients. Further large sample clinical studies are warranted to analyze the correlation between BPV and CSA-AKI.

**Graphical abstract:**

**Figure:**
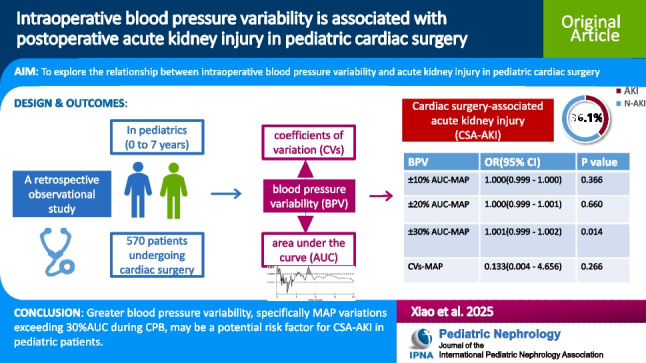
A higher resolution version of the Graphical abstract is available as Supplementary information

**Supplementary Information:**

The online version contains supplementary material available at 10.1007/s00467-025-06659-8.

## Introduction

China ranks among the nations with the highest incidence of congenital heart disease (CHD) globally. Annually, over 200,000 new cases of congenital heart disease are reported, with up to 85% necessitating surgical intervention [1, 2]. According to the *White Paper on Heart Surgery and Extracorporeal Circulation in China* 2022 [3], the number of CHD operations accounted for 25.8% of the total cardiovascular surgery in China, of which 58.6% were in pediatric patients. Additionally, the trend towards younger patient age and increasing surgical complexity poses significant challenges to perioperative management. Thus, optimizing cardiopulmonary bypass (CPB) management strategies and minimizing the incidence of perioperative complications in vital organs are crucial for improving pediatric prognosis.

Cardiac surgery-associated acute kidney injury (CSA-AKI) is the most common complication following cardiac surgery [4, 5]. The incidence of CSA-AKI varies by age and type of surgery, with about 5 to 45% in adults, while it ranges from 40 to 64% in children specifically [5, 6]. CSA-AKI is associated with increased mortality, prolonged mechanical ventilation, intensive care unit stay, and hospitalization [7–12]. Numerous factors are implicated in postoperative CSA-AKI, such as younger age, cyanotic lesions, longer CPB duration, and blood pressure fluctuation. However, the risk factors of CSA-AKI in the pediatric population remain not well-established [13]. The renal perfusion priority strategy during resuscitation proposes that kidneys are particularly vulnerable to hypoxia and hypoperfusion, and normal renal function is largely dependent on stable blood pressure [14]. Existing studies have shown that blood pressure variability (BPV) may be a risk factor for AKI. In a single-center retrospective observational study, 7504 adult patients undergoing coronary artery bypass graft surgery were included to analyze the correlation of systolic blood pressure (SBP) variation and mortality within 30 days. The results showed that the larger coefficients of variations (CVs) of SBP may be an important predictor of 30-day mortality after cardiac surgery [15]. Another study involving 3687 adults undergoing cardiac surgeries indicated that increased SBP variability was linked to 30-day mortality and kidney failure development, with surgery phase-specific correlations observed [16]. At present, BPV has not been extensively investigated in children, and some studies have only included non-cardiac surgeries, with varied results. A retrospective observational study found that larger BPV was associated with AKI in infants ≤ 12 months, but there was no relationship in children > 12 months [17]. Therefore, further research is needed to determine the correlation between BPV and CSA-AKI in pediatric cardiac surgery and the differences in different pediatric age groups. In order to explore the safe fluctuation range of blood pressure during CPB in pediatric cardiac surgery patients this study mainly included those under 7 years.

The objectives of this observational study were twofold: (1) to analyze the risk factors of CSA-AKI in children under 7 years and explore the relationship between intraoperative BPV and CSA-AKI in pediatric cardiac surgery patients; (2) to preliminarily explore the safe fluctuation range of BP during CPB in children under 7 years of age. The findings of this study will provide a corresponding theoretical basis for optimal BP management and CSA-AKI prevention in pediatric cardiac surgery.

## Methods and materials

### Patient population

A retrospective observational review of pediatric patients aged 7 years or younger undergoing cardiac surgery with CPB at West China Hospital of Sichuan University between 2019 and 2022 was conducted. The study was approved by the Clinical Research Ethics Committee of West China Hospital of Sichuan University (IRB number: No. 2021(1129); approval date: September 18, 2021). The requirement for informed consent was waived due to the retrospective nature of the analysis.

Children were excluded if they had severe preoperative renal insufficiency (evidenced by preoperative dialysis or a preoperative creatinine value greater than 3.0 mg/dL), had used nephrotoxic drugs prior to surgery, had received extracorporeal life support before surgery, had a history of radiotherapy or chemotherapy, were undergoing heart transplantation, required emergent surgery, or had incomplete clinical data.

### Anesthesia and CPB main protocol

General anesthesia was induced with midazolam 0.05 mg/kg, cisatracurium 0.1 to 0.2 mg/kg, sufentanil 0.5 to 1 ug/kg, and propofol 2 to 2.5 mg/kg. The trachea was intubated, and mechanical ventilation started to achieve an end tidal carbon dioxide tension of 35 to 45 mmHg. Anesthesia was maintained with continuous infusion of propofol or inhalation of sevoflurane until the end of surgery. Midazolam, sufentanil, and cisatracurium were given as needed.

A standard CPB with a disposable hollow-fiber membrane oxygenator (Affinity Pixie, Medtronic, Minneapolis, MN) and a roller pump (Stockert-5, Sorin Group, Munchen, Germany) were selected. The pump circuit was primed with 150 to 200 mL acetated Ringers solution, 1 to 2 units of packed red blood cells, 10 g albumin, 1.25 mL/kg 20% mannitol, and 10 to 20 mL 5% sodium bicarbonate. Cold blood cardioplegia (ratio of crystalloid to blood is 1:4) at a dose of 30 mL/kg was used for all patients. Cardioplegia was repeated with a half dose every 20 to 25 min during surgery. All patients were treated with mild hypothermic CPB (32–34 °C). During CPB, the targeted pump flow rate was maintained between 2.6 and 3.2 L/min/m^2^, with a mean arterial pressure (MAP) of 30 to 50 mmHg and a hematocrit of 25 to 30%. Blood gases were measured every 15 min. After the cardiac surgical procedure and aortic unclamping, the heart was defibrillated if sinus rhythm did not resume spontaneously. After weaning from CPB, modified ultrafiltration was routinely performed, and the hematocrit was maintained at 30 to 35%. Protamine was then used to reverse the effect of heparin. Postoperatively, all patients were transported intubated to the pediatric intensive care unit (PICU) to recover.

### BPV measurement

*BPV* [18] is a continuous variable that describes blood pressure variation, which can be described by standard deviation (*SD*), *CV*s, average real variation (*ARV*), and AUC. *SD* reflects the distribution of all *BP* means, regardless of the mean; while *CV*s are *SD* divided by the mean *BP*. *ARV* describes mean of the absolute differences between consecutive *BP* measurements. As a measure of discrete variability, *ARV* depends on the time sequence of measurements and takes into account the differences between individual consecutive measurements. AUC reflects the integral of the magnitude and duration of the deviation from a certain threshold.

Before surgery, baseline blood pressures were measured daily when children were awake and quiet in the ward. The intraoperative blood pressure included *SBP*, diastolic blood pressure (DBP), and MAP pre- and post-CPB, as well as MAP during CPB were recorded every 5 min. The BPV was described in the following two ways: (1) *CV*s [16] were calculated as *SD* divided by the mean of the values of blood pressure in the stage of pre- and post-CPB of systolic, diastolic, and MAP, as well as MAP during CPB, which reflected the degree of BPV; (2) deviation of the MAP during CPB from the baseline MAP was expressed as the AUC [18], which was calculated by multiplication of amplitude and duration of blood pressure deviation from baseline blood pressure. The baseline MAP was defined as the mean value MAP measured in the ward and during awake quiet or mild sedation prior to induction of anesthesia. The AUC deviation of ± 10%, ± 20%, and ± 30% from the baseline MAP during CPB were defined as ± 10% AUCm, ± 20% AUCm, and ± 30% AUCm, respectively.

### CSA-AKI definition

The CSA-AKI was defined as any decrease in estimated creatinine clearance (eCrCl) [19] > 25% from baseline (obtained within 1 week before surgery) to peak value within the first 7 days after surgery using the pediatric risk, injury, failure, loss, and end-stage kidney diseases (pRIFLE) criterion [13]. The stages of CSA-AKI were classified as follows: risk stage (decrease in eCrCl > 25% from baseline), injury stage (decrease in eCrCl > 50% from baseline), failure stage (decrease in eCrCl > 75% from baseline or eCrCl < 3 5 mL/min/1.73 m^2^), loss stage (duration of failure > 4 weeks), and end-stage (continuous failure > 3 months). It is important to note that the diagnosis of AKI did not consider urine output, as it is often affected by modified ultrafiltration and diuretic use.

### Data collection

Demographic and clinical data were collected from patients’ electronic medical records, including preoperative comorbidities, anesthetic records, surgery-related data, and clinical outcomes. Preoperative data included preoperative congenital complications, preoperative vasoactive drug use, congenital heart surgery risk assessment grade (RACHS-1), previous surgical history, left ventricular ejection fraction, and baseline blood pressure. Laboratory examination including serum creatinine (Scr), eCrCl, preoperative serum cystatin C, preoperative hemoglobin concentration (Hb) was also recorded. Surgery‐related data such as type of operation, duration of operation, CPB time, aortic cross-clamping time, intraoperative peak value of vasoactive inotropic score (VIS), intraoperative fluid input and output, and blood transfusion were documented. Clinical outcomes including CSA-AKI, PICU stay, postoperative mechanical ventilation time, length of hospital stay, incidence of low cardiac output syndrome (LCOS), liver insufficiency and pulmonary infection, and 30-day all-cause mortality, were recorded.

### Statistical analysis

Statistical analysis was performed using SPSS26.0. The measurement data were tested by Shapiro–Wilk for normality. For data adhering to a normal distribution, the results were presented as *mean* ± *SD*, and group comparisons were made using the *T*-test. Conversely, for non-normally distributed data, values were expressed as the median and interquartile range (*IQR*), with the Mann–Whitney *U* test employed for between-group comparisons. Categorical data were expressed in frequencies and percentages (%) and analyzed using the chi-square test or Fisher’s exact probability method for group comparisons. Univariate analysis was performed for all indicators of the children before and during operation. Chi-square test or Fisher’s exact test was used for the difference analysis between categorical variables, and *t*-test or Wilcoxon signed rank sum test was used for the difference analysis between continuous variables. Multivariate logistic regression analysis was used to analyze CSA-AKI related risk factors. All factors with *P* < 0.1 in the results of univariate analysis of preoperative and intraoperative data were included in the binary logistic regression analysis model as independent variables, and the occurrence of CSA-AKI was taken as the dependent variable. Spearman correlation was used to diagnose collinearity among independent variables. All tests were bilateral, and *P* < 0.05 was considered statistically significant. Subgroup comparison of BPV during cardiac surgery was conducted between AKI group and non-AKI group across different age groups (≤ 1 year old, 1–3 years old, and 3–7 years old).

## Results

### Participant’s characteristics

Among 757 cardiac surgical cases performed between 2019 and 2022, a total of 570 children met the inclusion criteria. Patients without CPB (*n* = 12), undergoing emergent surgery (*n* = 165), and with missing clinical data (*n* = 10) were excluded. The cohort comprised 289 males (50.7%) and 281 females (49.3%). The median weight was 10 kg (*IQR* 7–14), and the median height was 80.0 cm (*IQR* 66.7–95.0). A total of 26 patients (4.6%) presented with congenital complications preoperatively.

**Fig. 1:**
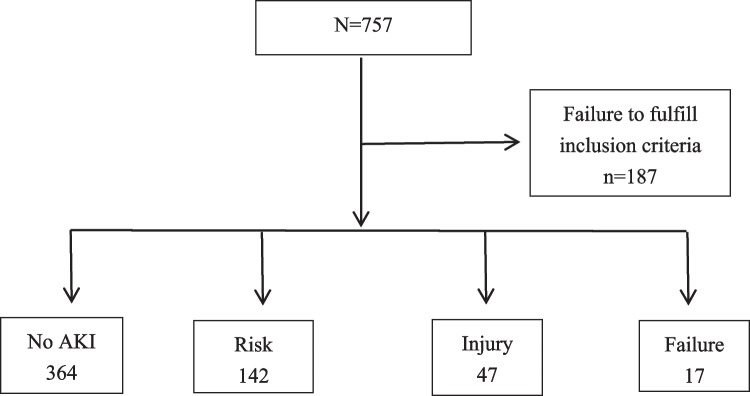
Patients with and without acute kidney injury (AKI) according to AKI presentation. Clinical course of patients was according to the renal pediatric risk, injury, failure, loss of renal function, and end-stage renal disease class

The overall incidence of CSA-AKI in this pediatric population was 36.1% (206 patients), among which, 68.9% at risk stage, 22.8% at injury stage, 8.3% at failure stage; none was in the loss or end-stage categories) (Fig. 1). The age distribution of CSA-AKI cases was 51.0% in the 0–1-year group, 32.5% in the 1–3-year group, and 16.5% in the 3–7-year group.

### Comparison between AKI group and non-AKI group

In the AKI group, males accounted for a larger proportion (*P* = 0.011), as did more congenital complications (*P* = 0.019), and younger age (*P* < 0.001). Regarding intraoperative data, children who developed CSA-AKI had longer CPB time, cross-clamping time, and surgical time, required more intraoperative blood infusion, and had higher VIS peak value and a higher ratio of RACHS-1 grade ≥ 3. The preoperative profile and intraoperative data of the AKI group and non-AKI group are detailed in Table 1.

**Table 1:** Demographics, preoperative profile, and intraoperative details of both cohorts

Variables	Non-AKI group (*n* = 364)	AKI group (*n* = 206)	*P* value
Age (year)	2.1 (0.9–3.8)	1.0 (0.6–2.2)	** < 0.001**
Male (%)	170 (46.7%)	119 (57.8%)	**0.011**
Congenital complications (%)	11 (3.0%)	15 (7.3%)	**0.019**
LVEF < 50%	2 (0.5%)	8 (3.9%)	1.432
Hb (g/L)	126 (120.5–133.5)	128 (121.0–132.0)	0.904
Serum cystatin C (mg/L)	0.88 (0.82–1.00)	0.91 (0.86–0.98)	0.379
Scr (μmol/L)	33 (28–39)	25 (21–31)	0.445
eCrCl (mL/min/1.73 m^2^)	90.5 ± 12.4	95.7 ± 22.4	0.543
Operation time (min)	175 (145–210)	245 (188–310)	** < 0.001**
Aortic occlusion time (min)	54 (39–73)	84 (54–115)	** < 0.001**
CPB time (min)	76 (61–98)	124 (85–164)	** < 0.001**
VIS peak value	3 (2–5)	7 (3–10)	** < 0.001**
Infusion quantity (mL)	180 (100–300)	150 (100–270)	0.170
Urine volume (mL)	50 (40–80)	50 (20–120)	0.416
**RACHS-1 (%)**			
Grade 3–5	73(20.1%)	76(36.9%)	** < 0.001**
**Intraoperative blood infusion (%)**	350 (96.2%)	198 (96.1%)	0.982
Red blood cell	29 (8%)	37 (18%)	** < 0.001**
Plasma	27 (7.4%)	34 (16.5%)	**0.001**
Platelet	19 (5.2%)	35 (17.0%)	** < 0.001**

Values are median (interquartile range) or *n* (%). *P* < 0.05 was considered significant

*LVEF* left ventricular ejection fraction, *eCrCl* estimated creatinine clearance, *Hb* preoperative, *Scr* serum creatinine, *RACHS-1* the risk adjustment in congenital heart surgery-1 method, *CPB* cardiopulmonary bypass, *VIS* variance index score

The significance of the bold emphasis is to visually show the variables with statistical differences

Intraoperative blood pressure between AKI group and non-AKI group showed no statistical difference in SBP, DBP, and MAP pre-, during, and post-CPB (Table 2). Similarly, there were no significant differences in CVs of blood pressure before, during, and after CPB between the two groups. However, ± 30% AUCm during CPB was higher in the AKI group (*P* = 0.038) (Table 2).

**Table 2:** Comparison of intraoperative blood pressure and BPV between AKI group and non-AKI group

BP (mmHg)	Non-AKI group (*n* = 364)	AKI group (*n* = 206)	*P* value
Pre-CPB			
SBP	90.8 ± 6.2	91.5 ± 7.4	0.486
DBP	52.3 ± 6.2	52.1 ± 7.3	0.829
MAP	62.0 ± 6.1	62.3 ± 6.4	0.485
During CPB			
MAP	45.9 ± 5.4	46.0 ± 6.7	0.701
Post-CPB			
SBP	84.7 ± 8.5	85.2 ± 9.6	0.698
DBP	48.3 ± 9.4	48.4 ± 9.2	0.806
MAP	61.1 ± 5.8	60.9 ± 5.8	0.692
BPV			
Pre-CPB CVs, median (*IQR*)			
SBP-CVs	0.071(0.049–0.107)	0.067(0.049–0.096)	0.418
DBP-CVs	0.011(0.069–0.157)	0.102(0.072–0.156)	0.890
MAP-CVs	0.083(0.055–0.111)	0.083(0.053–0.125)	0.627
CVs during CPB, median (*IQR*)			
MAP-CVs	0.179(0.144–0.204)	0.168(0.140–0.199)	0.118
Post-CPB CVs, median (*IQR*)			
SBP-CVs	0.066(0.044–0.103)	0.074(0.048–0.106)	0.217
DBP-CVs	0.097(0.070–0.133)	0.101(0.070–0.145)	0.623
MAP-CVs	0.069(0.042–0.098)	0.066(0.042–0.089)	0.497
AUCm during CPB, median (*IQR*)			
± 10% AUCm	386.16(272.24–510.09)	357.13(242.68–513.17)	0.162
± 20% AUCm	185.61(84.16–291.24)	174.63(67.145–291.95)	0.696
± 30% AUCm	30.95(0–138.25)	61.97(0–187.68)	**0.038**

Values are median (interquartile range) or *n* (%). *P* < 0.05 was considered significant

*CPB* cardiopulmonary bypass, *SBP* systolic blood pressure, *DBP* diastolic blood pressure, *MAP* mean arterial pressure, *CVs* pressure coefficients of variation, *AUCm* area under curve where mean arterial pressure deviates from baseline mean arterial pressure during cardiopulmonary bypass

The significance of the bold emphasis is to visually show the variables with statistical differences

Postoperatively, children in the AKI group were significantly more likely to develop LCOS (5.3% vs. 0.5%, *P* = 0.001), liver insufficiency (95.1% vs. 75.8%, *P* < 0.001), and pulmonary infection (50.0% vs. 14.6%, *P* < 0.001). They also had longer durations of mechanical ventilation (76 (12–150) vs. 5 (2–15) hours, *P* < 0.001), PICU stays (7 (4–12) vs. 2 (2–5) days, *P* < 0.001) and hospitalization (16 (11–21) vs. 8 (7–11) days, *P* < 0.001). However, the 30-day all-cause mortality rates were comparable between the two groups (Table 3).

**Table 3:** Postoperative outcomes in AKI group and non-AKI group

Variables	Non-AKI group (*n* = 364)	AKI group (*n* = 206)	*P* value
Hospital stays (day)	8 (7–11)	16 (11–21)	** < 0.001**
Postoperative mechanical ventilation time (h)	5 (2–15)	76 (12–150)	** < 0.001**
PICU time (day)	2 (2–5)	7 (4–12)	** < 0.001**
Pulmonary infection (%)	53 (14.6%)	103 (50.0%)	** < 0.001**
Liver insufficiency (%)	276 (75.8%)	196 (95.1%)	** < 0.001**
LCOS (%)	2 (0.5%)	11 (5.3%)	**0.001**
30-day all-cause mortality (%)	2(0.5%)	8(3.9%)	0.634

Values are median (interquartile range) or *n* (%). *P* < 0.05 was considered significant

*AKI* acute kidney injury, *PICU* pediatric intensive care unit, *LCOS* low cardiac output syndrome

The significance of the bold emphasis is to visually show the variables with statistical differences

### Univariate and multivariate analyses

Preoperative and intraoperative data were included in the univariate analysis. On univariate analysis, age (*P* < 0.001), male sex (*P* = 0.011), preoperative congenital complications (*P* = 0.023), RACHS-1 grade ≥ 3 (*P* < 0.001), time of operation (*P* < 0.001), aortic cross-clamp time (*P* < 0.001), CPB time (*P* < 0.001), peak value of VIS during operation (*P* < 0.001), intraoperative blood infusion (*P* < 0.001), and ± 30% AUCm (*P* = 0.014) showed statistically significant differences (Table 4). The risk variables with an explanatory *P* < 0.10 at the univariate step were tested with a multivariable approach.

**Table 4:** Univariable and multivariable analysis for predictors of CSA-AKI

Variables	*OR*	95% *CI*	*P* value
Univariate analysis			
Age (year)	0.754	0.677–0.839	** < 0.001**
Male (%)	1.561	1.106–2.203	**0.011**
Congenital complications (%)	2.520	1.135–5.596	**0.023**
RACHS-1 ≥ 3 grade	0.429	0.293–0.629	** < 0.001**
Operation time (min)	1.011	1.008–1.013	** < 0.001**
Aortic occlusion time (min)	1.025	1.019–1.031	** < 0.001**
CPB time (min)	1.021	1.017–1.026	** < 0.001**
VIS peak value	1.170	1.121–1.221	** < 0.001**
Infusion quantity (mL)	1.000	0.999–1.001	0.648
Urine volume (mL)	1.002	1.000–1.003	0.441
Intraoperative blood infusion (%)			
Red blood cell	2.529	1.503–4.254	** < 0.001**
Plasma	0.405	0.237–0.694	** < 0.001**
Platelet	0.269	0.149–0.480	** < 0.001**
Pre-CPB CVs			
SDP	0.079	0.002–3.700	0.196
DBP	1.912	0.145–25.124	0.622
MAP	1.857	0.064–53.641	0.718
In CPB CVs			
MAP	0.133	0.004–4.656	0.266
In CPB AUCm			
± 10% AUCm	1.000	0.999–1.000	0.366
± 20% AUCm	1.000	0.999–1.001	0.660
± 30% AUCm	1.001	1.000–1.002	**0.014**
Post-CPB CVs			
SBP	2.841	0.118–68.653	0.520
DBP	5.389	0.274–105.947	0.268
MAP	0.104	0.002–6.535	0.284
Multivariate analysis			
Age (year)	0.900	0.787–1.029	0.122
Male (%)	2.044	1.297–3.222	**0.002**
Congenital complications (%)	2.665	0.977–7.265	0.055
RACHS-1 ≥ 3 grade	0.510	0.307–0.846	**0.009**
CPB time (min)	1.022	1.007–1.037	**0.004**
VIS peak value	1.072	1.026–1.119	**0.002**
Red blood cell (%)	1.417	0.672–2.988	0.359
± 30% AUCm	1.000	0.998–1.001	0.610

Values are median (interquartile range) or *n* (%). *P* < 0.05 was considered significant

*CPB* cardiopulmonary bypass, *SBP* systolic blood pressure, *DBP* diastolic blood pressure, *MAP* mean arterial pressure, *CVs* pressure coefficients of variation, *AUCm* area under curve where mean arterial pressure deviates from baseline mean arterial pressure during cardiopulmonary bypass, *VIS* variance index score, *RACHS-1* the risk adjustment in congenital heart surgery-1 method

The significance of the bold emphasis is to visually show the variables with statistical differences

The various factors identified in the univariate analysis were tested for intercorrelation. There were significant correlations between operation time, aortic cross-clamping time, and CPB time, and between intraoperative plasma infusion, platelet infusion, and red blood cell infusion. Considering that the correlation of CPB time and intraoperative RBC infusion to CS-AKI is much stronger, we therefore included the CPB time and intraoperative RBC infusion in the multivariable models. After correction for the other explanatory variables, male gender (*OR* = 2.044, 95% *CI* 1.297–3.222, *P* = 0.002), RACHS-1 classification ≥ 3 (*OR* = 0.510, 95% *CI* 0.307–0.846, *P* = 0.009), longer CPB time (*OR* = 1.022, 95% *CI* 1.007–1.037, *P* = 0.004) and high intraoperative VIS peak value (*OR* = 1.072, 95% *CI*: 1.026–1.119, *P* = 0.002) were independent risk factors for CSA-AKI in children. However, ± 30% AUCm (*P* = 0.610) was not an independent risk factor for CSA-AKI (Table 4).

### Comparison of BPV in different pediatric age groups

The differences of ± 10% AUCm, ± 20% AUCm, and ± 30% AUCm in CPB were compared between AKI group and non-AKI group at different ages (≤ 1 year old, 1–3 years old, and 3–7 years old), and the results showed that there were no statistical differences in ± 10% AUCm and ± 20% AUCm at different ages (*P* > 0.05). There is no statistically significant difference in ± 30% AUCm between the AKI group and the non-AKI group (*P* > 0.05) in ages of 1–3, but the ± 30% AUCm in CPB of the AKI group is significantly greater than that of the non-AKI group (*P* = 0.031) in ages of 3–7, showing a statistically significant difference, as shown in Table 5.

**Table 5:** Comparison of BPV in pediatric with different ages

Year ≤ 1 (*n* = 217)			
BPVs	Non-AKI group (*n* = 112)	AKI group (*n* = 105)	*P* value
**Pre-CPB CVs, media (*IQR*)**			
SBP-CVs	0.068 (0.041–0.119)	0.067 (0.052–0.095)	0.745
DBP-CVs	0.101 (0.054–0.151)	0.096 (0.077–0.156)	0.325
MAP-CVs	0.081 (0.057–0.111)	0.082 (0.051–0.119)	0.809
**In CPB CVs, media (*IQR*)**			
MAP-CVs	0.181 (0.144–0.220)	0.165 (0.142–0.199)	0.151
**Post-CPB CVs, media (*IQR*)**			
SBP-CVs	0.065 (0.046–0.102)	0.080 (0.056–0.115)	0.088
DBP-CVs	0.095 (0.069–0.134)	0.101 (0.070–0.138)	0.991
MAP-CVs	0.071 (0.050–0.108)	0.065 (0.042–0.090)	0.119
± 10% AUCm, media (*IQR*)	338.00 (237.24–487.11)	313.68 (195.32–468.00)	0.509
± 20% AUCm, media (*IQR*)	166.26 (79.00–338.17)	183.00 (63.00–301.74)	0.734
± 30% AUCm, media (*IQR*)	105.34 (0–282.75)	93.00 (0–266.00)	0.702
**1 < year ≤ 3 (*n* = 181)**			
BPVs	Non-AKI group (*n* = 114)	AKI group (*n* = 67)	*P* value
Pre-CPB CVs, media (*IQR*)			
SBP-CVs	0.073 (0.049–0.092)	0.065 (0.046–0.094)	0.317
DBP-CVs	0.115 (0.077–0.170)	0.104 (0.064–0.162)	0.414
MAP-CVs	0.083 (0.053–0.115)	0.092 (0.063–0.131)	0.182
In CPB CVs, media (*IQR*)			
MAP-CVs	0.181 (0.145–0.203)	0.168 (0.136–0.194)	0.170
Post-CPB CVs, media (*IQR*)			
SBP-CVs	0.066 (0.041–0.107)	0.057 (0.042–0.099)	0.337
DBP-CVs	0.093 (0.071–0.128)	0.104 (0.076–0.148)	0.201
MAP-CVs	0.069 (0.045–0.098)	0.063 (0.042–0.089)	0.351
± 10% AUCm, media (*IQR*)	371.29 (232.46–469.71)	351.16 (265.29–471.79)	0.860
± 20% AUCm, media (*IQR*)	173.84 (57.28–244.55)	150.00 (56.21–251.05)	0.644
± 30% AUCm, media (*IQR*)	10.58 (0–83.72)	11.00 (0–97.00)	0.939
**3 < year ≤ 7 (*n* = 172)**			
BPVs	non-AKI group (n = 138)	AKI group (n = 34)	*P* value
**Pre-CPB CVs, media (*IQR*)**			
SBP-CVs	0.072 (0.054–0.103)	0.079 (0.050–0.095)	0.776
DBP-CVs	0.107 (0.069–0.152)	0.109 (0.080–0.162)	0.590
MAP-CVs	0.083 (0.054–0.110)	0.078 (0.056–0.126)	0.923
**In CPB CVs, media (*IQR*)**			
MAP-CVs	0.111 (0.094–0.173)	0.108 (0.086–0.163)	0.833
**Post-CPB CVs, media (*IQR***)			
SBP-CVs	0.065 (0.043–0.091)	0.078 (0.049–0.103)	0.411
DBP-CVs	0.104 (0.066–0.134)	0.091 (0.067–0.139)	0.814
MAP-CVs	0.062 (0.033–0.087)	0.069 (0.048–0.087)	0.430
± 10% AUCm, media (*IQR*)	439.11 (323.68–603.07)	428.68 (361.89–661.47)	0.489
± 20% AUCm, media (*IQR*)	194.71 (114.79–293.37)	239.05 (144.74–388.74)	0.367
± 30% AUCm, media (*IQR*)	18.21 (0–103.62)	104.26 (0–199.11)	**0.031**

Values are median (interquartile range) or *n* (%). *P* < 0.05 was considered significant

*CPB* cardiopulmonary bypass, *SBP* systolic blood pressure, *DBP* diastolic blood pressure, *MAP* mean arterial pressure, *CVs* pressure coefficients of variation, *AUCm* area under curve where mean arterial pressure deviates from baseline mean arterial pressure during cardiopulmonary bypass

The significance of the bold emphasis is to visually show the variables with statistical differences

## Discussion

AKI is one of the most common complications after cardiac surgery in pediatric CPB and is closely associated with postoperative outcome [5, 20, 21]. Our study results show that the incidence of CSA-AKI in pediatric patients under 7 years of age was 36.1% (51.0% were aged 0–1 year, 32.5% aged 1–3 years, and 16.5% aged 3–7 years). This study demonstrated that longer CPB time, RACHS-1 grade ≥ 3, and high intraoperative peak of VIS were risk factors associated with CSA-AKI. However, there was no significant association between CVs for SBP, DBP, and MAP and CSA-AKI during any period of surgery. In addition, the AUC of ± 10%, ± 20%, or ± 30% deviation from the baseline MAP during the bypass phase was not significantly associated with CSA-AKI.

The incidence of CSA-AKI varies across different age groups due to the immaturity of organ function in pediatric patients at various developmental stages, combined with complex cardiac malformations resulting from congenital heart disease, rendering them more susceptible to a range of pathophysiological effects [22]. There are great differences in risk factors of CSA-AKI at different developmental stages. Existing studies have shown that the risk factors leading to the occurrence of pediatric CSA-AKI can be divided into two categories, namely renal factors and extrarenal factors. The renal factors include decreased perfusion blood flow, decreased glomerular filtration rate, and use of nephrotoxic drugs. Extrarenal factors can be divided into patient factors, hemodynamic factors, and inflammatory factors [23]. Patient factors [8] include young age, low body weight, and RACHS-1 grade ≥ 3. Hemodynamic factors [7] include long-term CPB bypass, low cardiac output syndrome, postoperative use of high dose, and long-term vasoactive drugs, postoperative ECMO assistance, long-term mechanical ventilation, and early postoperative fluid overload.

In this study, the results show that male gender, RACHS-1 grade ≥ 3, long CPB time, and high peak intraoperative VIS score were independent risk factors for the development of pediatric CSA-AKI. This is consistent with the findings of previous studies. The study conducted by Blinder et al. [8] on 430 small infants aged 3 months demonstrated that a higher RACHS-1 grade, younger age, more complex cardiac malformations, elevated baseline creatinine levels, utilization of CPB, and longer duration of CPB were associated with an increased risk of CSA-AKI. The study conducted by Li et al. [6] on 311 pediatric patients undergoing cardiac surgery revealed that CSA-AKI was independently associated with RACHS-1 grade, age, duration of mechanical ventilation, surgery time, and ICU factors. A meta-analysis [24] showed that age was significantly associated with CSA-AKI in CHD surgery, and the incidence of CSA-AKI in patients < 1 month old was higher than that in other age groups. For studies with long CPB time, Park et al. [25] reported that the incidence of CSA-AKI in infants was significantly increased at CPB time > 120 min; while Hirano et al. [26] believed that 90 min of CPB was an independent risk factor for CSA-AKI in infants.

The pathogenesis of CSA-AKI is mainly related to perioperative renal hypoperfusion and imbalance of oxygen supply and demand [5, 27–29]. The perioperative hemodynamic disturbance and changes in vascular tension during CPB may even decrease renal blood flow by up to 30% [30, 31]. Intraoperative blood pressure instability may be an important factor leading to postoperative AKI [27, 28]. Thus, maintaining blood pressure within an appropriate threshold range and preventing renal hypoperfusion and oxygen insufficiency are important for preventing CSA-AKI. Because blood pressure is a continuous variable, it is difficult to evaluate quantitatively, so the BPV [18] is often used to measure its stability and variability. Studies of blood pressure management during cardiac surgery in adults have shown that increased intraoperative BPV is associated with adverse postoperative outcomes, increased incidence of kidney failure, and mortality at 30 days after surgery [16]. However, the physiological and pathological characteristics of pediatric patients are different from those of adults, and the influence of BPV on pediatric CSA-AKI needs further study.

In this study, *CV*s and AUC were used to describe BPV in pediatric cardiac surgery and analyze the correlation between intraoperative BPV and CSA-AKI. The results show that there were no correlations between *CV*s of SBP, DBP, MAP, and the occurrence of CSA-AKI. In bivariate analysis, ± 30% AUCm of BPV during CPB was associated with increased risk of AKI, but with no significance in multivariable analysis. A retrospective cohort [32] of children aged 1 to 17 years found that increased systolic and MAP-CVs were significantly associated with AKI, while measures of diastolic BPV were not associated with AKI in the multivariable model. A post-hoc analysis [33] of data from 3 prospective, open-label, and randomized clinical trials (ECLIPSE trials) in sixty-one medical centers in the USA, which included cardiac surgery patients ≥ 18 years, aimed to study the impact of perioperative BPV on health resource utilization after cardiac surgery. The results showed that increased perioperative SBP-AUC is associated with delayed extubation and increased postoperative length of stay. A prospective observational study [34] included patients aged 18 years or older and investigated the relationship between short-term BPV and incidence of AKI in critically ill patients. The conclusion was that SBP-ARV correlated with the incidence of AKI and a weak association was also found between *ARV* and hospital mortality in critically ill patients. A systematic review [18] of 11 articles analyzed the impact of intraoperative BPV on the risk of postoperative adverse outcomes in adult non-cardiac surgery, and a relationship between higher intraoperative CV, ARV and postoperative complications was observed in 5 studies [35–39]. A probable protective effect of higher BPV was found on the risk of postoperative complications in three studies [40–42]. One study [43] observed a U-shaped relationship between MAP-ARV and postoperative complications; however, there was no association between the variable and the outcome in the other two studies [36, 44]. A study [45] retrospectively examining 600 patients found that preoperative DBP-CV and intraoperative SBP-CV were independent risk factors for ninety-day postoperative negative outcomes. A retrospective, observational, cohort study [46], of those undergoing cardiac surgery requiring CPB, showed that CV-SBP/MAP were not predictive of mortality and kidney failure. A retrospective study [47] assessed the patients referred for elective coronary artery bypass graft with the use of ECC, the main conclusions of which were that DBP is more labile than SBP, and BPV is the greatest during CPB. A retrospective, single-center study [16], indicated increasing SBP-CV was associated with 30-day mortality and development of kidney failure in cardiac surgery. A cross-sectional study [48] showed that SBP-CV was independently associated with AKI in aortic dissection surgery.

The stratification of BPV and CSA-AKI in pediatric cardiac surgery has not been conducted based on different age groups. A retrospective cohort study [32] examining the associations between BPV and AKI in patients aged 1 to 17 years found that SBP and MAP variability were significantly associated with AKI, however, the subgroup age analysis of this study only included statistical analysis of children ≥ 13 years old or < 13 years old, without detailed analysis by age group. A retrospective chart review [17] was conducted on children aged 21 or younger who underwent cardiac surgery with CPB, which found that higher BPV was associated with higher risk of postoperative AKI in infants. However, the age stratification was only divided into less than or more than 12 months. In our study, more detailed age stratification analysis was carried out for children less than 7 years old, including 1 month to 1 year old, 1 year old to 3 years old, and 3 to 7 years old. Therefore, the results of this study may have more clinical significance.

The prognosis of patients is significantly influenced by CSA-AKI. The results of a meta-analysis [49] indicated that the in-hospital mortality for AKI patients was 7–22 times higher compared to non-AKI patients. Additionally, AKI patients experienced longer durations of mechanical ventilation, PICU stays, and hospitalization than non-AKI patients. Sethi et al. [9] found that CSA-AKI increased the use of vasoactive drugs and prolonged mechanical ventilation, PICU, and hospital stay. Li et al. [6] showed that the duration of postoperative mechanical ventilation, PICU duration and mortality during hospitalization were independent risk factors for the development of CSA-AKI. Graziani et al. [50] showed that there were higher intraoperative vasoactive drug use, higher VIS scores, longer mechanical ventilation and hospital stay, more bleeding, and more neurological complications in AKI patients compared with non-AKI patients. The results of this study showed that postoperative mechanical ventilation, PICU stay and hospital stay were longer in the AKI group, and incidence of important postoperative complications (including LCOS, hepatic insufficiency, and pulmonary infection) were higher than in the non-AKI group, which was consistent with previous studies. Different from some previous studies, in which 30-day all-cause mortality was regarded as an important influencing factor of cardiac surgery-related mortality, there was no group difference in 30-day all-cause mortality between AKI and non-AKI groups in this study. This difference may be related to the relatively small sample size and short study period in this study, requiring further analysis of more large and multicenter clinical studies.

This study has some limitations. First, the AUC used in the BPV analysis of this study only described the blood pressure integral outside the deviation threshold range; however, statistical analysis of below and above baseline was not performed separately. Second, this study is a retrospective study with the presence of unmeasured potential confounders. Third, this study used only creatinine to define the occurrence of AKI, which may result in a missed diagnosis of CSA-AKI. Simultaneously combining novel biomarkers of kidney injury, such as serum cystatin C, neutrophil gelatinase-associated lipocalin, and liver-type-fatty acid-binding protein, may be helpful to identify more patients at an earlier stage. Fourth, this study did not further compare the differences of BPV in different types of congenital heart disease surgery. Fifth, only the amount of fluid in and out during operation was retrospectively analyzed, and the whole perioperative input and output were not obtained.

In conclusion, greater BPV, specifically MAP variations exceeding ± 30% AUC during CPB, may be a potential risk factor for CSA-AKI in pediatric cardiac surgery. Further clinical studies are warranted to analyze the correlation between BPV and CSA-AKI across different age groups in pediatric cardiac surgery.

## Supplementary Information

Below is the link to the electronic supplementary material.

Graphical abstract (PPTX 247 KB)

## Data Availability

The data that support the findings of this study are available from the corresponding author upon reasonable request.

## References

[1] Zhao QM, Liu F, Wu L, Ma XJ, Niu C, Huang GY (2019) Prevalence of congenital heart disease at live birth in China. J Pediatr 204:53–58. 10.1016/j.jpeds.2018.08.040 30270157 10.1016/j.jpeds.2018.08.040

[2] Sun PF, Ding GC, Zhang MY, He SN, Gao Y, Wang JH (2017) Prevalence of congenital heart disease among infants from 2012 to 2014 in Langfang, China. Chin Med J (Engl) 9:1069–1073. 10.4103/0366-6999.204923 10.4103/0366-6999.204923 PMC5421177 28469102

[3] Chinese Society of Extracorporeal Circulation (2022) White book of Chinese cardiovascular surgery and extracorporeal circulation in 2021. Chin J ECC 20:196–199

[4] Yu Y, Li C, Zhu S, Jin L, Hu Y, Ling X, Miao C, Guo K (2023) Diagnosis, pathophysiology and preventive strategies for cardiac surgery-associated acute kidney injury: a narrative review. Eur J Med Res 28:45. 10.1186/s40001-023-00990-2 36694233 10.1186/s40001-023-00990-2 PMC9872411

[5] Wang Y, Bellomo R (2017) Cardiac surgery-associated acute kidney injury: risk factors, pathophysiology and treatment. Nat Rev Nephrol 11:697–711. 10.1038/nrneph.2017.119 10.1038/nrneph.2017.119 28869251

[6] Li S, Krawczeski CD, Zappitelli M, Devarajan P, Thiessen-Philbrook H, Coca SG, Kim RW, Parikh CR, TRIBE-AKI Consortium (2011) Incidence, risk factors, and outcomes of acute kidney injury after pediatric cardiac surgery: a prospective multicenter study. Crit Care Med 39:1493–1499. 10.1097/CCM.0b013e31821201d3 21336114 10.1097/CCM.0b013e31821201d3 PMC3286600

[7] Alabbas A, Campbell A, Skippen P, Human D, Matsell D, Mammen C (2013) Epidemiology of cardiac surgery-associated acute kidney injury in neonates: a retrospective study. Pediatr Nephrol 28:1127–1134. 10.1007/s00467-013-2454-3 23519522 10.1007/s00467-013-2454-3

[8] Blinder JJ, Goldstein SL, Lee VV, Baycroft A, Fraser CD, Nelson D, Jefferies JL (2012) Congenital heart surgery in infants: effects of acute kidney injury on outcomes. J Thorac Cardiovasc Surg 2:368–374. 10.1016/j.jtcvs.2011.06.021 10.1016/j.jtcvs.2011.06.021 21798562

[9] Sethi SK, Kumar M, Sharma R, Bazaz S, Kher V (2015) Acute kidney injury in children after cardiopulmonary bypass: risk factors and outcome. Indian Pediatr 3:223–226. 10.1007/s13312-015-0611-4 10.1007/s13312-015-0611-4 25848999

[10] Piggott KD, Soni M, Decampli WM, Ramirez JA, Holbein D, Fakioglu H, Blanco CJ, Pourmoghadam KK (2015) Acute kidney injury and fluid overload in neonates following surgery for congenital heart disease. World J Pediatr Congenit Heart Surg 6:401–406. 10.1177/2150135115586814 26180155 10.1177/2150135115586814

[11] Ueno K, Shiokawa N, Takahashi Y, Nakae K, Kawamura J, Imoto Y, Kawano Y (2020) Kidney disease: improving global outcomes in neonates with acute kidney injury after cardiac surgery. Clin Exp Nephrol 24:167–173. 10.1007/s10157-019-01805-7 31677063 10.1007/s10157-019-01805-7

[12] Lysak N, Bihorac A, Hobson C (2017) Mortality and cost of acute and chronic kidney disease after cardiac surgery. Curr Opin Anaesthesiol 30:113–117. 10.1097/ACO.0000000000000422 27841788 10.1097/ACO.0000000000000422 PMC5303614

[13] Zhang Y, Wang B, Zhou XJ, Guo LJ, Zhou RH (2022) Nadir oxygen delivery during pediatric bypass as a predictor of acute kidney injury. Ann Thorac Surg 113:647–653. 10.1016/j.athoracsur.2021.01.026 33524358 10.1016/j.athoracsur.2021.01.026

[14] Wang X, Chao Y, Liu L (2022) Shock resuscitation - the necessity and priority of renal blood perfusion assessment. Aging Dis 13:1056–1062. 10.14336/ad.2022.0105 35855346 10.14336/AD.2022.0105 PMC9286909

[15] Aronson S, Stafford-Smith M, Phillips-Bute B, Shaw A, Gaca J, Newman M (2010) Intraoperative systolic blood pressure variability predicts 30-day mortality in aortocoronary bypass surgery patients. Anesthesiology 113:305–312. 10.1097/ALN.0b013e3181e07ee9 20571360 10.1097/ALN.0b013e3181e07ee9

[16] Jinadasa SP, Mueller A, Prasad V, Subramaniam K, Heldt T, Novack V, Subramaniam B (2018) Blood pressure coefficient of variation and its association with cardiac surgical outcomes. Anesth Analg 127:832–839. 10.1213/ANE.0000000000003362 29624524 10.1213/ANE.0000000000003362

[17] Fishbein JE, Barone M, Schneider JB, Meyer DB, Hagen J, Bakar A, Grammatikopoulos K, Sethna CB (2022) Blood pressure variability during pediatric cardiac surgery is associated with acute kidney injury. Pediatr Nephrol 37:871–879. 10.1007/s00467-021-05234-1 34436673 10.1007/s00467-021-05234-1

[18] Putowski Z, Czok M, Krzych LJ (2022) The impact of intraoperative blood pressure variability on the risk of postoperative adverse outcomes in non-cardiac surgery: a systematic review. J Anesth 36:316–322. 10.1007/s00540-022-03035-w 35028755 10.1007/s00540-022-03035-w PMC8967760

[19] Ranucci M, Johnson I, Willcox T, Baker RA, Boer C, Baumann A, Justison GA, de Somer F, Exton P, Agarwal S, Parke R, Newland RF, Haumann RG, Buchwald D, Weitzel N, Venkateswaran R, Ambrogi F, Pistuddi V (2018) Goal-directed perfusion to reduce acute kidney injury: a randomized trial. J Thorac Cardiovasc Surg 156(1918–1927):e2. 10.1016/j.jtcvs.2018.04.045 10.1016/j.jtcvs.2018.04.045 29778331

[20] Khuong JN, Wilson TG, Iyengar AJ, d’dekem Y (2021) Acute and chronic kidney disease following congenital heart surgery: a review. Ann Thorac Surg 112:1698–1706. 10.1016/j.athoracsur.2020.10.054 33310148 10.1016/j.athoracsur.2020.10.054

[21] Zappitelli M, Parikh CR, Kaufman JS, Go AS, Kimmel PL, Hsu CY, Coca SG, Chinchilli VM, Greenberg JH, Moxey-Mims MM, Ikizler TA, Cockovski V, Dyer AM, Devarajan P, ASsessment, Serial Evaluation, and Subsequent Sequelae in Acute Kidney Injury (ASSESS-AKI) Investigators (2020) Acute kidney injury and risk of CKD and hypertension after pediatric cardiac surgery. Clin J Am Soc Nephrol 15:1403–1412. 10.2215/CJN.00150120 32948644 10.2215/CJN.00150120 PMC7536759

[22] Davies LK (1999) Cardiopulmonary bypass in infants and children: how is it different? J Cardiothorac Vasc Anesth 13:330–345. 10.1016/s1053-0770(99)90274-4 10392688 10.1016/s1053-0770(99)90274-4

[23] Leballo G, Chakane PM (2020) Cardiac surgery-associated acute kidney injury: pathophysiology and diagnostic modalities and management. Cardiovasc J Afr 31:205–212. 10.5830/CVJA-2019-069 32555928 10.5830/CVJA-2019-069 PMC8762796

[24] Li D, Niu Z, Huang Q, Sheng W, Wang T (2020) A meta-analysis of the incidence rate of postoperative acute kidney injury in patients with congenital heart disease. BMC Nephrol 21:350. 10.1186/s12882-020-02005-2 32807107 10.1186/s12882-020-02005-2 PMC7433101

[25] Park SK, Hur M, Kim E, Kim WH, Park JB, Kim Y, Yang JH, Jun TG, Kim CS (2016) Risk factors for acute kidney injury after congenital cardiac surgery in infants and children: a retrospective observational study. PLoS ONE 11:e0166328. 10.1371/journal.pone.0166328 27832187 10.1371/journal.pone.0166328 PMC5104485

[26] Hirano D, Ito A, Yamada A, Kakegawa D, Miwa S, Umeda C, Chiba K, Takemasa Y, Tokunaga A, Ida H (2017) Independent risk factors and 2-year outcomes of acute kidney injury after surgery for congenital heart disease. Am J Nephrol 46:204–209. 10.1159/000480358 28858859 10.1159/000480358

[27] Newland RF, Baker RA (2017) Low oxygen delivery as a predictor of acute kidney injury during cardiopulmonary bypass. J Extra Corpor Technol 49:224–230 29302112 PMC5737422

[28] de Somer FMJ, Bryan MR, Aloisio T, Van Nooten GJ, Ranucci M (2011) O2 delivery and CO2 production during cardiopulmonary bypass as determinants of acute kidney injury: time for a goal-directed perfusion management? Crit Care 15:R192. 10.1186/cc10349 21831302 10.1186/cc10349 PMC3387634

[29] Magruder JT, Weiss SJ, DeAngelis KG, Haddle J, Desai ND, Szeto WY, Acker MA, Magruder JT, Acker A, Koprivanac M, Grimm JC, Patel SJ, Weiss SJ, DeAngelis KG, Haddle J, Ottemiller S, Cevasco M, Desai ND, Szeto WY, Acker MA (2022) Correlating oxygen delivery on cardiopulmonary bypass with Society of Thoracic Surgeons outcomes following cardiac surgery. J Thorac Cardiovasc Surg 164:997–1007. 10.1016/j.jtcvs.2020.12.008 33485654 10.1016/j.jtcvs.2020.12.008

[30] Massoth C, Zarbock A (2021) Diagnosis of cardiac surgery-associated acute kidney injury. J Clin Med 10:3664. 10.3390/jcm10163664 34441960 10.3390/jcm10163664 PMC8397056

[31] Vives M, Hernandez A, Parramon F, Estanyol N, Pardina B, Munoz A, Alvarez P, Hernandez C (2019) Acute kidney injury after cardiac surgery: prevalence, impact and management challenges. Int J Nephrol Renovasc Dis 12:153–166. 10.2147/IJNRD.S167477 31303781 10.2147/IJNRD.S167477 PMC6612286

[32] Nugent JT, Ghazi L, Yamamoto Y, Bakhoum C, Wilson FP, Greenberg JH (2023) Hypertension, blood pressure variability, and acute kidney injury in hospitalized children. J Am Heart Assoc 12:e029059. 10.1161/jaha.122.029059 37119062 10.1161/JAHA.122.029059 PMC10227226

[33] Aronson S, Levy JH, Lumb PD, Fontes M, Wang Y, Crothers TA, Sulham KA, Navetta MS (2014) Impact of perioperative blood pressure variability on health resource utilization after cardiac surgery: an analysis of the ECLIPSE Trials. J Cardiothorac Vasc Anesth 28:579–585. 10.1053/j.jvca.2014.01.004 24726635 10.1053/j.jvca.2014.01.004

[34] Xie Z, Liao X, Yin W, Kang Y, Guo J, Lu M (2017) Relationship between short-term blood pressure variability and incidence of acute kidney injury in critically ill patients. Kidney Blood Press Res 42:1238–1246. 10.1159/000485927 29248933 10.1159/000485927

[35] Neuner B, Weiss B, Wulfekammer T, Radtke F, Franck M, Spies C (2016) GW27-e0549 Absolute values of blood pressure fluctuation as an independent risk factor for postoperative delirium: secondary, exploratory analysis of the randomized controlled “Surgery depth of anaesthesia and cognitive outcome”- (SuDoCO) trial. J Am Coll Cardiol 68:C185. 10.1016/j.jacc.2016.07.699

[36] Li J, Zhao Y, Zhao M, Cao P, Liu X, Ren H, Zhang D, Zhang Y, Wang R, Zhao J (2020) High variance of intraoperative blood pressure predicts early cerebral infarction after revascularization surgery in patients with Moyamoya disease. Neurosurg Rev 43:759–769. 10.1007/s10143-019-01118-z 31203482 10.1007/s10143-019-01118-z

[37] Park S, Lee HC, Jung CW, Choi Y, Yoon HJ, Kim S, Chin HJ, Kim M, Kim YC, Kim DK, Joo KW, Kim YS, Lee H (2020) Intraoperative arterial pressure variability and postoperative acute kidney injury. Clin J Am Soc Nephrol 15:35–46. 10.2215/CJN.06620619 31888922 10.2215/CJN.06620619 PMC6946069

[38] Wiorek A, Krzych LJ (2019) Intraoperative blood pressure variability predicts postoperative mortality in non-cardiac surgery-a prospective observational cohort study. Int J Environ Res Public Health 16:4380. 10.3390/ijerph16224380 31717505 10.3390/ijerph16224380 PMC6888597

[39] Cai YH, Wang HT, Zhou JX (2016) Perioperative predictors of extubation failure and the effect on clinical outcome after infratentorial craniotomy. Med Sci Monit 22:2431–2438. 10.12659/msm.899780 27404044 10.12659/MSM.899780 PMC4944551

[40] James LA, Levin MA, Lin HM, Deiner SG (2019) Association of preoperative frailty with intraoperative hemodynamic instability and postoperative mortality. Anesth Analg 128:1279–1285. 10.1213/ANE.0000000000004085 31094800 10.1213/ANE.0000000000004085

[41] Prasad V, Toschi N, Canichella A, Marcellucci M, Coniglione F, Dauri M, Guerrisi M, Heldt T (2015) Intraoperative hemodynamics predict postoperative mortality in orthotopic liver transplantation. Annu Int Conf IEEE Eng Med Biol Soc 2015:989–992. 10.1109/embc.2015.7318530 26736430 10.1109/EMBC.2015.7318530

[42] Levin MA, Fischer GW, Lin HM, McCormick PJ, Krol M, Reich DL (2015) Intraoperative arterial blood pressure lability is associated with improved 30 day survival. Br J Anaesth 115:716–726. 10.1093/bja/aev293 26395645 10.1093/bja/aev293

[43] Mascha EJYD, Weiss S, Sessler DI (2015) Intraoperative mean arterial pressure variability and 30-day mortality in patients having noncardiac surgery. Anesthesiology 123:79–91. 10.1097/ALN.0000000000000686 25929547 10.1097/ALN.0000000000000686

[44] Zevallos CB, Dai B, Dandapat S, Quispe-Orozco D, Holcombe A, Ansari S, Farooqui M, Derdeyn CP, Samaniego EA, Ortega-Gutierrez S (2021) Greater intraprocedural systolic blood pressure and blood pressure variability are associated with contrast-induced neurotoxicity after neurointerventional procedures. J Neurol Sci 420:117209. 10.1016/j.jns.2020.117209 33187680 10.1016/j.jns.2020.117209

[45] Benolken MM, Meduna AE, Klug MG, Basson MD (2021) Preoperative and intraoperative blood pressure variability independently correlate with outcomes. J Surg Res 266:387–397. 10.1016/j.jss.2021.04.027 34087623 10.1016/j.jss.2021.04.027 PMC8338749

[46] Packiasabapathy S, Prasad V, Rangasamy V, Popok D, Xu X, Novack V, Subramaniam B (2020) Cardiac surgical outcome prediction by blood pressure variability indices Poincaré plot and coefficient of variation: a retrospective study. BMC Anesthesiol 20:56. 10.1186/s12871-020-00972-5 32126969 10.1186/s12871-020-00972-5 PMC7055104

[47] Lazzeroni D, Camaiora U, Castiglioni P, Bini M, Garibaldi S, Geroldi S, Moderato L, Brambilla L, Brambilla V, Parati G, Coruzzi P (2020) In-hospital day-by-day systolic blood pressure variability during rehabilitation: a marker of adverse outcome in secondary prevention after myocardial revascularization. J Hypertens 9:1729–1736. 10.1097/hjh.0000000000002489 10.1097/HJH.0000000000002489 32516294

[48] Li Y, Zheng T, Zhu J, Chen Y, Chen Y, Sun S (2023) Variability of blood pressure and risk of postoperative acute kidney injury in patients undergoing surgery for acute aortic dissection: a 11-year single-center study. J Clin Hypertens 25:463–469. 10.1111/jch.14654 10.1111/jch.14654 PMC10184486 36974374

[49] Van den Eynde J, Rotbi H, Gewillig M, Kutty S, Allegaert K, Mekahli D (2021) In-hospital outcomes of acute kidney injury after pediatric cardiac surgery: a meta-analysis. Front Pediatr 9:733744. 10.3389/fped.2021.733744 34540775 10.3389/fped.2021.733744 PMC8446539

[50] Graziani MP, Moser M, Bozzola CM, Galvez HM, Irman Garrido J, Alvarez PG, Fernie ML (2019) Acute kidney injury in children after cardiac surgery: risk factors and outcomes. A retrospective, cohort study. Arch Argent Pediatr 117:e557–e567. 10.5546/aap.2019.eng.e557 31758882 10.5546/aap.2019.eng.e557

