# Supplementary material for: Relationship between intraoperative blood pressure variability and postoperative acute kidney injury in pediatric cardiac surgery
Source: Pediatr Nephrol. 2025 Jan 27;40(6):2071–81. doi: 10.1007/s00467-025-06659-8 (PMC12031837; doi:10.1007/s00467-025-06659-8)
Supplement: Supplementary file 1 — Graphical abstract (PPTX 247 KB) [file 467_2025_6659_MOESM1_ESM.pptx]

## Slide 1
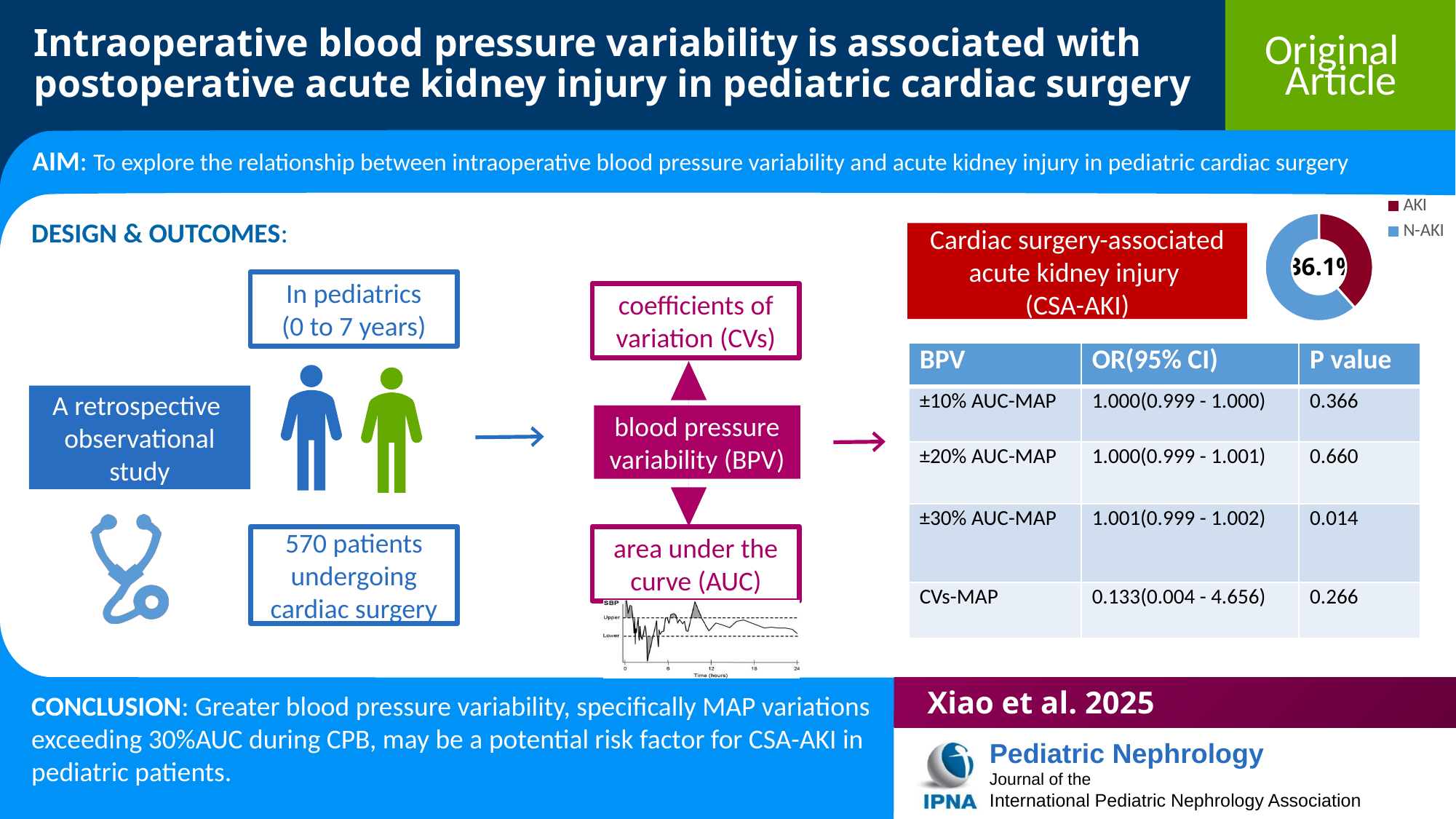

Intraoperative blood pressure variability is associated with postoperative acute kidney injury in pediatric cardiac surgery
AIM: To explore the relationship between intraoperative blood pressure variability and acute kidney injury in pediatric cardiac surgery
### Chart
| Category | 发生率 |
|---|---|
| AKI | 38.5 |
| N-AKI | 61.5 |DESIGN & OUTCOMES:
Cardiac surgery-associated acute kidney injury
(CSA-AKI)
36.1%
In pediatrics
(0 to 7 years)
coefficients of variation (CVs)
| BPV | OR(95% CI) | P value |
| --- | --- | --- |
| ±10% AUC-MAP | 1.000(0.999 - 1.000) | 0.366 |
| ±20% AUC-MAP | 1.000(0.999 - 1.001) | 0.660 |
| ±30% AUC-MAP | 1.001(0.999 - 1.002) | 0.014 |
| CVs-MAP | 0.133(0.004 - 4.656) | 0.266 |
A retrospective observational study
blood pressure variability (BPV)
area under the curve (AUC)
570 patients
undergoing cardiac surgery
Xiao et al. 2025
CONCLUSION: Greater blood pressure variability, specifically MAP variations exceeding 30%AUC during CPB, may be a potential risk factor for CSA-AKI in pediatric patients.
